# Glutathione Peroxidases: An Emerging and Promising Therapeutic Target for Pancreatic Cancer Treatment

**DOI:** 10.3390/antiox13111405

**Published:** 2024-11-16

**Authors:** Paula Iglesias-Matesanz, Carlos Lacalle-Gonzalez, Carlos Lopez-Blazquez, Michael Ochieng’ Otieno, Jesus Garcia-Foncillas, Javier Martinez-Useros

**Affiliations:** 1 Genomics and Therapeutics in Prostate Cancer Group, I+12 Biomedical Research Institute, 28041 Madrid, Spain; iglesias.imas12@h12o.es; 2 START Madrid-FJD, Hospital Fundacion Jimenez Diaz, 28040 Madrid, Spain; carlos.lacalle@quironsalud.es; 3 Translational Oncology Division, OncoHealth Institute, Health Research Institute Fundación Jimenez Diaz, Fundación Jimenez Díaz University Hospital, Universidad Autonoma de Madrid (IIS-FJD, UAM), 28040 Madrid, Spain; carlos.lblazquez@gmail.com (C.L.-B.); mikeotson@yahoo.com (M.O.O.); jgfoncillas@quironsalud.es (J.G.-F.); 4 Medical Oncology Department, Fundación Jimenez Diaz University Hospital, 28040 Madrid, Spain; 5 Area of Physiology, Department of Basic Health Sciences, Faculty of Health Sciences, Rey Juan Carlos University, 28922 Madrid, Spain

**Keywords:** pancreatic cancer, ferroptosis, GPx4, glutathione peroxidases, drug resistance

## Abstract

Glutathione peroxidases (GPxs) are a family of enzymes that play a critical role in cellular redox homeostasis through the reduction of lipid hydroperoxides to alcohols, using glutathione as a substrate. Among them, GPx4 is particularly of interest in the regulation of ferroptosis, a form of iron-dependent programmed cell death driven by the accumulation of lipid peroxides in the endoplasmic reticulum, mitochondria, and plasma membrane. Ferroptosis has emerged as a crucial pathway in the context of cancer, particularly pancreatic cancer, which is notoriously resistant to conventional therapies. GPx4 acts as a key inhibitor of ferroptosis by detoxifying lipid peroxides, thereby preventing cell death. However, this protective mechanism also enables cancer cells to survive under oxidative stress, which makes GPx4 a potential druggable target in cancer therapy. The inhibition of GPx4 can trigger ferroptosis selectively in cancer cells, especially in those that rely heavily on this pathway for survival, such as pancreatic cancer cells. Consequently, targeting GPx4 and other GPX family members offers a promising therapeutic strategy to sensitize pancreatic cancer cells to ferroptosis, potentially overcoming resistance to current treatments and improving patient outcomes. Current research is focusing on the development of small-molecule inhibitors of GPx4 as potential candidates for pancreatic cancer treatment.

## 1. Introduction

Pancreatic ductal adenocarcinoma (PDAC) is a highly lethal cancer, primarily due to its late diagnosis. The latest statistics report 66,440 estimated new cases and 51,750 estimated deaths for both sexes in the United States in 2024 [1]. The lethality of PDAC is largely due to the absence of early detection and screening methods. The five-year survival rate is significantly higher when tumors are detected early, at less than 2 cm, reaching up to 50% for tumors <2 cm and 100% for tumors <1 cm. Therefore, due to late detection, when metastasis has often occurred, the survival rate drops dramatically to 3% [2]. Surgical resection offers the only potential cure; however, it is viable for only 15–20% of patients at diagnosis, often hindered by a vascular invasion. Even with surgery, recurrence rates reach over 80% systemically and over 20% locally. Hence, treatments like chemotherapy, radiotherapy, and combined modalities are utilized before and after surgery, though they provide limited survival benefits [3]. Gemcitabine has been the cornerstone of PDAC chemotherapy, traditionally used alone but now often combined with nab-paclitaxel. This combination extends the median overall survival (OS) from 6.7 months with monotherapy to 8.5 months in combination [4]. FOLFIRINOX, a multi-drug regimen composed of folinic acid, 5-FU, irinotecan, and oxaliplatin, offered a better OS of 21.6 months compared to 12.8 months with gemcitabine [5]. Recent advancements include the NAPOLI-3 trial, which introduced NALIRIFOX, a modified FOLFIRINOX regimen with liposomal irinotecan. It showed an improved median OS (11.1 months) and progression-free survival (PFS) (7.4 months) over the gemcitabine/nab-paclitaxel combination. Given these compelling findings, NALIRIFOX is emerging as the preferred chemotherapy option for patients with metastatic PDAC [6]. Despite significant success in other cancers, immunotherapy has not been effective for PDAC, likely due to the tumor’s complex microenvironment [7]. Targeted therapies have shown limited success, particularly those targeting *KRAS* G12C mutations, which are present in only 1–2% of PDAC cases. Sotorasib, targeting this mutation, showed a 21% response rate and median OS of 6.9 months, but limited patient applicability and short duration of response are major drawbacks [8]. Therefore, the ongoing challenge of PDAC underscores the urgent need for research into novel treatments, which emphasizes the necessity for breakthroughs specifically tailored to pancreatic cancer.

In this concern, GPxs have provided a new insight for cancer treatment due to their role in protecting cells from oxidative stress by reducing peroxides. GPxs are often dysregulated in several types of cancers like lung, breast, and colorectal and can influence tumor progression and chemoresistance by modulating reactive oxygen species (ROS) levels [9]. In this review, we compile the evidence that supports GPx proteins as a potential target for novel treatment strategies against PDAC.

## 2. Glutathione Peroxidases Are Crucial for Oxidative Metabolism

GPxs are crucial enzymes in cellular antioxidant defense, reducing hydroperoxides to water and alcohols using glutathione (GSH) as a reductant [10]. This enzyme family comprises eight isoforms (GPx1-8) that are differentiated by the presence of either selenocysteine or cysteine at their active sites [10]. GPx1 is the isoform with the highest specificity for GSH due to the unique combination of amino acids present at its catalytic site [10]. As a result, it is considered the primary contributor to the catalysis of H_2_O_2_, and it is ubiquitously expressed in both the cytosol and mitochondria of nearly all tissues [11]. GPx2 expression is mainly restricted to the gastrointestinal tract, where it protects against the absorption of peroxides naturally found in food [10]. GPx3, secreted in plasma as a glycoprotein, functions extracellularly to protect against systemic oxidative stress by neutralizing ROS generated by surface lipoxygenases [12]. Unlike GPx1-3, GPx4 is a monomer, similar to GPx7 and GPx8, while the other GPx enzymes are tetramers [9]. GPx4 exists in three different isoforms: cytosolic (cGPx4), mitochondrial (mGPx4), and sperm nuclear (snGPx4). While the cytosolic form is ubiquitous, mGPx4 and snGPx4 are specific to the testes [13]. This enzyme not only reduces standard peroxides but is also capable of reducing lipid peroxides already incorporated into cellular membranes, thereby helping to maintain their integrity [14]. GPx4 is essential for cellular homeostasis, and its knockout results in embryonic lethality [15]. GPx5 is expressed in the epididymis, where it protects sperm from oxidative stress [13]. GPx6 is found in the olfactory epithelium and is believed to be involved in the metabolism of odorants. GPx7 is highly expressed in the preadipocytes of white adipose tissue and is involved in maintaining general redox homeostasis. Finally, GPx8 is concentrated in the lungs where it prevents hydroperoxides from leaking from the endoplasmic reticulum [10].

## 3. The Involvement of Glutathione Peroxidases in Ferroptosis and PDAC

Ferroptosis is a unique, non-apoptotic form of cell death driven by the iron-dependent accumulation of lethal ROS, leading to the peroxidation of membrane lipids [16]. The primary ROS source in ferroptosis is the Fenton reaction, catalyzed by iron ions, although the specific roles of iron and ROS in ferroptosis are still being elucidated. ROS are a double-edged sword in cancer biology. On one hand, a moderate ROS level can promote DNA damage, fostering carcinogenesis and tumor progression [17]; on the other hand, high or excessive ROS levels can trigger programmed cell death mechanisms [10]. Recently, antioxidant enzymes have been acknowledged for having a dual role in cancer: they act as tumor-suppressor genes and as oncogenes during tumorigenesis. However, during tumor progression, these enzymes help cancer cells evade ROS-mediated cell death, aiding in their survival [18]. Specially, GPx4 is a central modulator of ferroptosis as it reduces lipid hydroperoxides to lipid alcohols, preventing the peroxidation of membrane lipids and maintaining membrane integrity [19]. GPx4 activity is contingent on GSH levels, with cysteine uptake (via the system Xc-antiporter) being crucial for GSH synthesis. Depletion of either GSH or cysteine can trigger ferroptosis in cancer cells, including PDAC [20].

Emerging research supports ferroptosis as a promising approach for PDAC treatment. Agents like zalcitabine induce ferroptosis by generating ROS, showing cytotoxic effects in PDAC cell lines [21]. Ferroptosis inducers may enhance chemotherapy sensitivity or overcome resistance, exemplified by chrysin, which boosts gemcitabine efficacy in PDAC [22]. The interplay between GPx4, ferroptosis, and chemotherapy resistance underscores the potential for combination therapies [23].

## 4. The Prognostic Significance of Glutathione Peroxidases in Cancer

GPxs are direct antioxidant enzymes that play an essential role in maintaining cellular homeostasis by protecting cells from oxidative damage. Their significance extends into the realm of cancer, where different isoforms of GPx have been linked to patient survival and prognosis across various cancer types (Table 1). Notably, there is currently no updated data on the prognostic role of GPx5 and GPx6 in cancer patients. The significance of various isoforms of GPx in prognosis across different cancer types underscores the need for future investigations in this field. Considering that the expression levels of different GPx isoforms can vary in a tissue-dependent manner, future patient-focused research should prioritize the development of a signature that encompasses all GPx isoforms. Identifying the most crucial isoform in a specific cancer type can pave the way for advancements in the therapeutic field for oncologic purposes. 

Remarkably, the extensive literature addressing the prognostic implications of GPxs in cancer, coupled with the limited evidence in the context of PDAC, presents a compelling avenue for further investigation and offers promising therapeutic opportunities for PDAC patients. 

## 5. The Prognostic Significance of Glutathione Peroxidases in PDAC

Antioxidant enzymes play a crucial role in cancer development, including PDAC. Chronic pancreatitis, a major risk factor for PDAC, is characterized by reduced antioxidant enzyme activity compared to healthy tissue. This reduction is even more pronounced in PDAC, suggesting a diminished capacity to counteract ROS, which indicates a potential progression from chronic pancreatitis to PDAC [48]. In this concern, GPx proteins appear as key mediators in ROS homeostasis, being GPx1-4 the most studied. 

**Figure 1:**
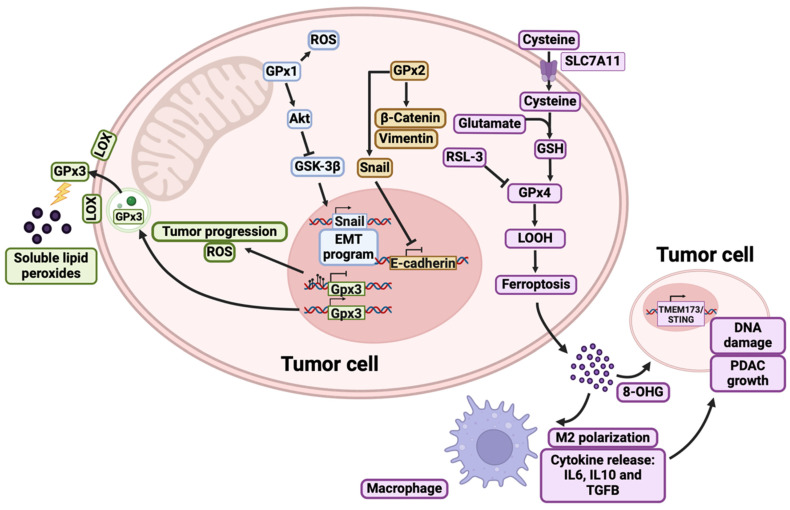
Molecular pathways of GPx1, GPx2, GPx3, and GPx4 in PDAC. In blue boxes are highlighted those pathways modulated by GPx1, which has been involved in chemoresistance [29]. The molecular pathway modulated by GPx2, which is associated with epithelial–mesenchymal transition (EMT), are highlighted in yellow boxes [49]. In green boxes are shown how GPx3 is a major regulator of ROS levels [12]. In purple boxes are highlighted how GPx4 is a master regulator of ferroptosis, having the ability to act on lipid hydroperoxides (LOOHs) and prevent lipid peroxidation of the cell membrane. On the contrary, when GPx4 is inhibited, the cell may enter ferroptosis and release signals to the extracellular matrix to activate other cells such as macrophages or other tumor cells that increase their tumor-prone phenotype [52].

Extensive research has focused on GPx1 due to its involvement in various diseases, not to mention the presence of gene polymorphisms that could serve as prognostic factors for cancer. The primary focus has been on the Pro198Leu gene polymorphism (rs1050450 C>T), although its susceptibility in different types of cancer is not yet fully understood [29]. GPx1 knockout (KO) mice exposed to normal oxidative stress exhibit no distinct phenotype. However, under severe stress conditions, these KO mice die even when supplemented with selenium [49]. This finding highlights the critical role of GPx1, as it cannot be compensated for by other selenoproteins under conditions of generalized stress [12]. Previous studies have reported that GPx1 expression levels gradually decrease in pancreatic cells, progressing from normal pancreatic tissue to chronic pancreatitis and ultimately to PDAC [48]. This downregulation suggests a potential tumor-suppressive role for GPx1 in this neoplasia. Moreover, increased expression of GPx1 has been shown to suppress the malignant phenotype both *in vivo* and in vitro (51% and 39% tumor cell growth suppression compared to controls, respectively) [50]. Conversely, the silencing of GPx1 promotes epithelial–mesenchymal transition (EMT) by activating the ROS-mediated Akt/GSK3β/Snail signaling axis in PDAC cells, leading to the induction of gemcitabine resistance [51] (Figure 1).

GPx2 is significantly overexpressed in PDAC tissues [53]. Protein and mRNA analyses have demonstrated that GPx2 upregulates β-catenin, vimentin, and Snail, while downregulating E-cadherin, in PDAC to promote EMT (Figure 1). Additionally, the silencing of GPx2 has been shown to reduce the expression of metalloproteinases (MMPs) MMP2 and MMP9 by downregulation of the Wnt pathway and inhibition of inflammation-mediated carcinogenesis, which are critical for cancer cell proliferation, migration, and invasion [54].

Regarding GPx3, it appears to function primarily as a systemic antioxidant; however, its expression varies across different tumors. While low levels of GPx3 are associated with a poor prognosis in several cancers, its upregulation in PDAC is linked to reduced ROS levels and increased chemoresistance together with superoxide dismutase (SOD1) and peroxidases (GPx2, thyroperoxidase, and myeloperoxidase) [55] (Figure 1).

GPx4 has not been directly studied as a prognostic marker in PDAC. In an in vitro study, it appeared to be upregulated in Panc-1 cancer stem cells (CSCs) compared with parental Panc-1 cells. Therefore, the elevated levels of GPx4 could be responsible for regulating oxidative homeostasis, maintaining the EMT program and maintaining the undifferentiated and, subsequently, more aggressive phenotype [56]. Interestingly, GPx4, further to being involved in lipid metabolism and the inhibition of ferroptosis, also plays a significant role in tumor vascularization. Schneider et al. demonstrated that the partial inactivation of GPx4 in mouse models leads to substantial changes in tumor vascular characteristics, like thinner diameters and markedly reduced vessel lumina. This fact suggested its involvement in tumor angiogenesis through the modulation of lipoxygenases-12 and 15 (Figure 1) [57].

Another pathway that is activated along with PDAC tumorigenesis by promoting cancer growth and metastasis via tumor microenvironment (TME) modulation is through TMEM173/STING. This pathway promotes ferroptosis in human PDAC in vitro leading to subsequent ROS production and lipid peroxidation by an increase in MFN1/2-dependent mitochondrial fusion [58]. Interestingly, depletion of TMEM173/STING protects against GPx4-depletion-induced neoplastic progression and reduces tumor-associated macrophages (Figure 1) [52].

## 6. GPx4 as a Potential Therapeutic Target in PDAC

The diverse roles of GPx isoforms in cancer highlight their potential as predictive markers. The variability in GPx expression across different cancer types and stages suggests that a comprehensive GPx signature could enhance patient stratification and treatment strategies. The identification and target of the most relevant GPx isoform in specific cancers could pave the way for new therapeutic approaches. In this concern, GPx4 has emerged as a vital regulator of ferroptosis, a form of programmed cell death characterized by lipid peroxidation. Its significant role in mitigating oxidative damage and maintaining cellular homeostasis has set it as a promising therapeutic target, particularly in PDAC. GPx4 is fundamental to the system Xc–/GSH/GPx4 axis, which protects cells from lipid peroxidation and subsequent ferroptosis [59]. Ferroptosis inducers (FINs) have been classified based on their mechanisms: class I FINs deplete glutathione (GSH), class II FINs directly inhibit GPx4, class III FINs can deplete GPx4 and coenzyme Q10 (CoQ10) via the squalene synthase–mevalonate pathway, and class IV FINs increase labile iron or oxidize iron to induce lipid peroxidation. Interestingly, key FINs like erastin, RSL-3, and ML-162 have been widely studied for their ability to modulate GPx4 and induce ferroptosis in PDAC [60]. Targeting GPx4 has been confirmed to be a potential treatment strategy against PDAC. While FINs, like erastin and RSL-3, demonstrate *in vitro* efficacy, their clinical development is hampered by their pharmacokinetics [61]. However, GSH depletion has been effective in inhibiting PDAC cell proliferation [62], and rapamycin has shown anti-cancer activity through GPx4 degradation [63]. Erastin inhibits cystine uptake, depleting GSH, while RSL-3 and ML-162 were previously thought to directly inhibit GPx4, but recent findings suggest they target thioredoxin reductase 1 (TXNRD1) instead [64]. Novel compounds such as oleanolic-acid-derived triterpenoids, e.g.,: CCDO-Me and benzothiazole compounds, have also been identified for their ability to induce apoptosis and inhibit SOD and GPx activities in PDAC cells [65,66]. Another compound, thiostrepton (TST), binds to STAT3, which inhibits GPx4 expression, while downregulation of the upstream Transcription Factor 2 (USF2) shows a reduction in GPx4 and solute carrier family 7 member 11 (SLC7A11) expression that increases lipid peroxidation, which sets USF2 as an important mediator in PDAC [67]. Recent studies have focused on combination therapies integrating FINs and traditional chemotherapy. MnFe2O4-loaded nanoparticles combined with gemcitabine showed increased ferroptosis in PDAC models [68]. Targeting the hexosamine biosynthetic pathway (HBP) with compounds like FR054 alongside erastin has promoted GSH depletion and ferroptosis [69].

However, cancer cells can develop resistance to ferroptosis. Studies in PDAC cells have shown resistance linked to the overexpression of nuclear factor erythroid 2-related factor 2 (Nrf2) and other factors like protein Tyrosine Phosphatase Mitochondrial 1 (PTPMT1) and ADP-ribosylation factor 6 (ARF6). PTPMT1 has been reported to be upregulated in PDAC and inhibits ferroptosis by suppressing the expression of acyl-CoA synthetase long chain family member 4 (ACSL4) and upregulating SLC7A11 *in vitro* [70]. ARF6 confers PDAC cells sensitivity to oxidative stress, due to RSL3-induced lipid peroxidation, and contributes to gemcitabine resistance via multiple pathways [71]. It is important to consider these compounds as a potential therapy in PDAC because they address a new paradigm in ferroptotic PDAC cell death. Future clinical trials must consider combination therapies including these novel compounds with standard chemotherapy due to its aptness to drive excessive and unendurable oxidative stress levels. Due to the paradoxical effects of reactive oxygen species (ROS) modulation, it is crucial to determine the metabolic state of cancer cells required to trigger ferroptosis. Cellular metabolic stress and the amount of ROS present in a cell vary according to its metabolic state; low levels are physiological, while intermediate levels can be protumorigenic since ROS react directly with DNA, allowing the accumulation of mutations within tumor cells. However, high levels are detrimental, as cellular damage cannot be adequately recovered by the remaining antioxidant enzymes, leading to cell death via ferroptosis [72]. This balance, where ROS must not exceed a certain threshold, forms the theoretical basis for GPx inhibition. Disrupting this equilibrium may drive cancer cells to a transition from an intermediate level, which is advantageous for their survival, to a toxic level triggering ferroptosis. In the context of advanced cancer, ferroptosis represents a potential therapeutic weapon; however, the role of GPx inhibition in carcinogenesis originating from precancerous lesions or healthy cells needs separate investigations.

Among the various isoenzymes, GPx4 is a monomer and is easy to inhibit. GPx4 exhibits a low protein expression across most tissues, achieving medium levels in thyroid glandular cells and enterocytes of the small intestine. Consequently, the toxicity profile should primarily be thyroidal and gastrointestinal, both of which are typically manageable in clinical practice [73]. In contrast, other isoenzymes such as GPx1 have a moderate to high expression in most tissues and function as tetramers, which makes effective inhibition with minimal toxicity rather challenging. The toxicity observed in embryos underscores the critical role of GPx4 in embryonic cells, akin to undifferentiated cancer cells, which are prevalent in advanced cancer. This fact reaffirms its utility while warning of its potential genotoxicity in humans.

Currently, there are no clinical trials assessing toxicity in humans. Nonetheless, cumulative evidence from animal studies suggests a potential toxicity. In the work by Yang J. et al., BALB/c nude mice were treated with RSL3, Cetuximab, or a combination of both, and all mice survived across all study arms with no serious reported toxicity [74]. Furthermore, in the study by Shengbiao L. et al., B-NDG mice were treated with RSL3 with no toxic deaths in the study [75]. Based on these data, the *in vivo* inhibition of GPx4 in mice, alongside the *in vitro* toxicity profile, should be further investigated in phase I clinical trials. Table 2 summarizes the main compounds to target GPx proteins and their direct targets to induce cell death by ferroptosis or apoptosis.

Other drugs like ophiopogonin-B induce ferroptosis in gastric cancer cells by inhibiting SLC7A11 and GPx4 expression [79]. Sanguinarine and Bufotalin promote GPx4 degradation, which enhances ferroptosis in non-small cell lung cancer [80,81]. Solasonine enables the induction of ferroptosis in hepatocellular carcinoma by the inhibition of GPx4 and glutathione synthetase [82]. Arsenic trioxide (ATO) has shown efficacy in inducing ferroptosis by blocking GPx4 activity in neuroblastoma [83].

GPx4’s role in the regulation of ferroptosis and oxidative damage makes it a compelling target for cancer therapy, especially in PDAC. Despite challenges in drug development and resistance, ongoing research and novel compound development offer promising avenues for targeting GPx4 to improve patients’ outcomes. The human safety evaluation of GPx4-targeted treatments warrants consideration for ongoing phase I clinical trials. This approach is particularly relevant since the inhibition exerts a promising preclinical activity. In the context of PDAC, it could present a valuable therapeutic opportunity for establishing especially a third-line treatment following NALIRIFOX or Gemcitabine plus nab-paclitaxel, where a standard of care is currently lacking to enhance response rates of conventional chemotherapy regimens by overcoming chemoresistance. However, it is crucial to assess the safety of combining GPx4 inhibition with chemotherapy in phase Ib/II clinical trials.

## 7. Glutathione Peroxidases Modulate the Immune Microenvironment in PDAC

**Figure 2:**
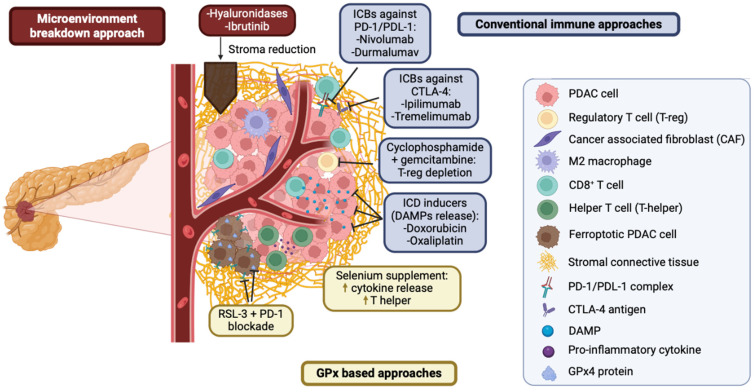
The tumor microenvironment of a PDAC and potential effects of GPx approaches. Red boxes show potential approaches to inhibit the TME, yellow boxes indicate GPx-based approaches, and blue boxes indicate conventional therapies to hamper the immune system. Arrows indicate activation and T bars denote repression.

PDAC is known for its unique TME, which features clusters of cancer cells and a dense stroma that constitutes about 80% of the tumor mass [84]. This stroma evolves as the tumor progresses, significantly impacting tumor growth, metastasis, immune evasion, and drug resistance. The dense stroma acts basically as a physical barrier that hampers vascularization and reduces drug penetration, impeding treatment to reach cells. For example, gemcitabine is more effective in lesions with a minimal stroma but struggles to penetrate those with high desmoplasia [85]. To improve drug delivery in PDAC, several strategies focusing on the TME have been proposed, e.g., hypoxia-activated pro-drugs, inhibitors of the Sonic Hedgehog pathway, or with hyaluronidase enzymes [86]. Hyaluronidase enzymes and fibrosis inhibitors like Ibrutinib have been developed to reduce the stromal barrier [86] (Figure 2. Red boxes).

PDAC is commonly called a “cold” tumor due to its low T-cell infiltration and immunosuppressive environment, driven by the presence of cancer-associated fibroblasts (CAFs) and hypoxia [87]. Immune evasion mechanisms in PDAC include limited tumor-associated antigens, downregulation of the major histocompatibility complex (MHC) molecules, and Fas/Fas ligand abnormalities, which lead to the apoptosis of T-effector cells and recruitment of immunosuppressive cells like TAMs, MDSCs, and Tregs [7]. Immunotherapies based on PD-1/PD-L1 or CTLA-4 blockade, such as Durvalumab/Nivolumab plus Tremelimumab/Ipilimumab, have limited effectiveness as single agents in PDAC (Figure 2. Blue boxes) [88]. However, their combination with gemcitabine has shown promising results, particularly in TGF-β-deficient patients. TGF-β plays a key role in the PDAC stroma promoting fibrosis and immune suppression, which can hinder immune cell infiltration and boost the efficacy of immunotherapy. In patients with TGF-β deficiency, the reduced stromal fibrosis and less immunosuppressive microenvironment allow for better immune cell infiltration and a more robust anti-tumor immune response, which enhance the effectiveness of immune checkpoint inhibitors combined with gemcitabine [86]. Furthermore, to promote anti-tumor immunity in PDAC, several strategies have been developed based on the depletion of Tregs, which are key contributors to immune suppression in the TME. Certain therapies such as doxorubicin, oxaliplatin, and radiation are used to induce immunogenic cell death, a process that releases damage-associated molecular patterns (DAMPs) and activates the immune system. These treatments can help to convert cold tumors into hot tumors, promoting the infiltration of immune cells into the tumor and boosting the immune response. This approach aims to overcome the immunosuppressive environment of PDAC, making the tumor more susceptible to immune therapies and improving their overall efficacy [89]. In a phase I/II trial, 17 patients with PDAC received treatment with gemcitabine, nab-paclitaxel, and pembrolizumab. The maximum tolerated dose was determined to be pembrolizumab 2 mg/kg every 21 days, gemcitabine 1000 mg/m^2^, and nab-paclitaxel 125 mg/m^2^ on days 1 and 8 every 21 days. Among 11 evaluable patients, the disease control rate was 100%, median PFS was 9.1 months, and overall survival was 15 months [90]. Lynch-syndrome-associated PDAC, characterized by microsatellite instability (MSI), may benefit from PD-1 blockade, as these tumors show better responses to immunotherapy [91]. The combination of gemcitabine with PD-1/PD-L1 antibodies has demonstrated significant anti-tumor effects in mouse models [92].

An exciting area of research in cancer therapy is focused on GPx4, particularly given its central role in ferroptosis (Figure 2. Yellow boxes). From the immune system point of view, selenium supplementation is known to enhance the activity of GPx4 and has shown an increased immune infiltration, particularly of T-helper cells, which are essential for the initiation and maintenance of effective immune responses [93]. This suggests that selenium could help to prime the immune system in PDAC by improving immune cell infiltration and enhancing anti-tumor immunity.

On the other hand, the combination of GPx4 inhibitors with PD-1 blockers has shown promising results in preclinical models, such as those in triple-negative breast cancer, where it increased CD8+ T cell infiltration and increased response rates [94]. The use of GPx4 inhibitors, such as RSL3, which induces ferroptosis, can disrupt the tumor’s redox balance and trigger cell death that enhances the release of tumor antigens (Figure 2. Left yellow box). When combined with gemcitabine-based chemotherapy and PD-1 blockade, this approach has been demonstrated to sensitize PDAC tumors to immune checkpoint inhibition *in vivo* [95]. The induction of ferroptosis helps to break immune tolerance, increase immune cell activity, and improve tumor visibility to the immune system, ultimately raising the therapeutic response to PD-1 blockade.

These findings suggest that GPx4 inhibition through strategies like selenium supplementation or the use of RSL3, in combination with gemcitabine and immune checkpoint inhibitors, offers a promising avenue for improving immune responses and overcoming the challenges of immune suppression and a fibrotic stroma in PDAC. These results justify further clinical investigations to explore GPx4 degradation as a potential therapeutic strategy to improve the efficacy of current PDAC treatments [95].

In summary, the modulation of the immune microenvironment in PDAC through a range of innovative strategies holds significant promise to improve the efficacy of immunotherapy in this highly challenging cancer. Key approaches include targeting the dense stromal barrier, which limits drug delivery and immune cell infiltration, as well as employing immune checkpoint blockades to reinvigorate exhausted immune cells. Additionally, the depletion of immunosuppressive cells, such as Tregs, can help overcome the tumor’s immune evasion mechanisms. The induction of ferroptosis through GPx4 inhibition has emerged as a novel strategy to promote tumor cell death and enhance immune system activation. Together, these strategies aim to reprogram the TME, enhance immune cell infiltration, and sensitize tumors to checkpoint inhibitors, potentially overcoming the barriers that have hindered the effective treatment of PDAC. Further clinical investigation is needed in the field of GPx4 inhibition, alone or in combination with gemcitabine, and immune checkpoint blockade. This is essential to unlock new therapeutic avenues for patients with PDAC.

## 8. Conclusions and Future Perspectives

The role of GPx proteins, particularly GPx4, in PDAC therapy has garnered significant attention due to their involvement in redox homeostasis and ferroptosis. GPx4 is critical to protect cells from lipid peroxidation; therefore, the inhibition of GPx4 leads to the accumulation of toxic lipid peroxides that trigger ferroptosis. Studies have shown that certain cancer cells, especially those resistant to traditional apoptosis-inducing therapies, are highly dependent on GPx4 for survival. 

The future treatment perspectives of GPx proteins are rather hopeful and multifaceted. As GPx4 is crucial for preventing lipid peroxidation and ferroptosis, its inhibitors (such as RSL3 and ML162) are being explored as potential therapeutic agents. Future treatments may combine GPx4 inhibitors with existing cancer therapies, such as immune checkpoint inhibitors or tyrosine kinase inhibitors. This approach could enhance the overall response rate of treatments by the exploitation of the increased oxidative stress within the TME. Synthetic lethality, where two non-lethal gene defects result in cell death, is also a propitious avenue. For example, the combination of GPx4 inhibition with drugs targeting mutations in *TP53* or *RAS* genes could selectively kill cancer cells without affecting normal cells. This approach is particularly relevant for cancers with known genetic mutations that already predispose them to oxidative stress. With the implementation of genetic screenings, treatments could be tailored to identify PDAC patients with specific mutations that make them more susceptible to GPx4 inhibition, which may improve outcomes. 

Since many cancers develop resistance to standard treatments, GPx4 inhibition could potentially reverse resistance in cancers that rely on redox balance for survival, making resistant cancers more susceptible to treatment. In this concern, future research will likely focus on developing more specific and potent inhibitors of GPx proteins. These new drugs would need to selectively target cancer cells to minimize damage to healthy tissues, which would enhance their therapeutic index. Innovations in drug delivery, including the use of nanotechnology, may improve the delivery and efficacy of GPx inhibitors, ensuring that they reach tumor cells more effectively and minimizing systemic toxicity. The inhibition of GPx proteins could make cancer cells more susceptible to therapies that increase oxidative stress, such as radiation therapy or certain chemotherapies, thus overcoming resistance. Although preclinical studies have shown satisfactory results, GPx4 inhibition in routine clinical practice may require careful consideration to ensure the selective eradication of cancer cells while limiting toxicity to normal tissues. Early-phase clinical trials are expected to explore the safety and efficacy of GPx4 inhibitors, particularly in combination with existing cancer therapies, focusing on treatment-refractory tumors. Therefore, translation of the preclinical efficacy of GPx inhibitors, either alone or in combination with other therapies, to clinical practice will be key only by keeping acceptable toxicity rates. 

In summary, the future perspectives for GPx proteins in PDAC treatment revolve around the selective inhibition of GPx4 to induce ferroptosis, the potential of synthetic lethality strategies, and the development of novel inhibitors that can be used in combination with existing therapies to overcome drug resistance and improve patient outcomes. These approaches are still in their early stages but hold significant promise to boost the effectiveness of the PDAC standard treatment.

## Figures and Tables

**Table 1:** Main oncologic features of glutathione peroxidases in different types of cancers.

GPx Isoform	Cancer Type	Association/Impact	PMID/Reference
GPx1	Gastric cancer	Elevated GPx1 levels correlate with poor patient outcomes.	[24]
	Kidney cancer	GPx1 expression is associated with aggressive tumor features and reduced survival.	[25]
	Acute myeloid leukemia (AML)	GPx1 impacts prognosis and survival rates in AML patients.	[26]
	Head and neck cancer	GPx1 expression contributes to tumor progression and patient survival.	[27]
	Low-grade glioma	GPx1 is linked to disease progression and survival outcomes.	[28]
	Clear cell renal carcinoma (ccRCC)	Upregulation correlates with advanced stages, metastasis, and shorter survival.	[25]
	Breast cancer	GPx1 polymorphisms, particularly Pro198Leu, are associated with increased cancer risk.	[29]
GPx2	Prostate cancer	High GPx2 levels are linked to poor prognosis.	[30]
	Hepatocellular carcinoma	GPx2 expression correlates with aggressive tumor characteristics.	[31]
	High-grade glioma	Elevated GPx2 expression worsens patient outcomes.	[32]
	Gastric cancer	Elevated GPx2 expression in tumors and lymphatic metastases correlates with aggressive tumor behavior.	[33]
GPx3	Thyroid cancer	GPx3’s role in prognosis is under investigation.	[34]
	Breast cancer	GPx3 expression is considered in determining patient survival.	[35]
	Cervical cancer	Lower GPx3 expression correlates with lymph node metastasis.	[36]
	Gallbladder cancer	Negative GPx3 expression is associated with reduced survival.	[37]
	Hepatocellular carcinoma	Low GPx3 levels predict poor outcomes.	[38]
GPx4	Epithelial ovarian cancer	GPx4 has prognostic value.	[39]
	Thyroid cancer	High GPx4 expression relates to disease advancement.	[40]
	Breast cancer	GPx4 levels are crucial for understanding breast cancer outcomes.	[41]
	Pan-cancer analysis	GPx4 expression is generally higher in tumor tissues compared to normal tissues across various cancers.	[42]
	Pancreatic ductal adenocarcinoma (PDAC)	GPx4’s role as a biomarker is under-explored, though it is suggested to regulate oxidative homeostasis and EMT in cancer stem cells.	[43]
GPx7	Gliomas	GPx7 is associated with poor prognosis.	[44]
GPx8	Gastric cancer	GPx8 is linked to unfavorable outcomes.	[45]
	Breast cancer	GPx8 is linked to unfavorable outcomes.	[46]
	Non-small cell lung cancer	GPx8 is linked to unfavorable outcomes.	[47]

**Table 2:** Main compounds to target the glutathione peroxidases under evaluation and their associated pathways.

Compound	Target	Mechanism	Cell Death	Reference
Erastin	SLC7A5 (Xc^−^ system)	GSH depletion by blocking cysteine entrance	Ferroptosis	[76]
RSL-3	GPx4 (TXNRD1)	Inhibition through active site binding	Ferroptosis	[60]
ML-162	GPx4 (TXNRD1)	Inhibition through active site binding	Ferroptosis	[64]
Rapamycin	GPx4	GPx4 protein degradation	Ferroptosis	[63]
CCDO-Me	Akt, Bcl-2, GPx4, mTOR and NF-κB	Anti-apoptotic protein inhibition	Apoptosis	[77]
Thiosteptron (TST)	STAT3	Binding to STAT3 and thus decreasing GPx4	Ferroptosis	[67]
USF2	SLC7A11 and GPx4	Increasing Fe^2+^ and thus lipid peroxidation in the cell	Ferroptosis	[78]
Manganese ferrite (nanoparticles) + gemcitabine	GSH	ROS production and GSH depletion	Ferroptosis	[68]
FR054 + erastin	PGM3	Glutamine metabolism disruption and GSH depletion	Ferroptosis	[69]

## Data Availability

Correspondence and requests for materials should be addressed to J. Martínez-Useros.
